# Snakes and Souks: Zoonotic pathogens associated to reptiles in the Marrakech markets, Morocco

**DOI:** 10.1371/journal.pntd.0011431

**Published:** 2023-07-19

**Authors:** Jairo Alfonso Mendoza-Roldan, Viviane Noll Louzada-Flores, Nouha Lekouch, Intissar Khouchfi, Giada Annoscia, Andrea Zatelli, Frédéric Beugnet, Julia Walochnik, Domenico Otranto

**Affiliations:** 1 Department of Veterinary Medicine, University of Bari, Valenzano, Italy; 2 Clinvet SA, Mohammedia, Morocco; 3 Boehringer Ingelheim Animal Health, Lyon, France; 4 Center for Pathophysiology, Infectiology and Immunology, Medical University of Vienna, Vienna, Austria; 5 Department of Pathobiology, Faculty of Veterinary Science, Bu-Ali Sina University, Hamedan, Iran; The University of Hong Kong, CHINA

## Abstract

The world-famous markets of Marrakech, also known in Arabic as souks, harbor a vast diversity of reptiles that are sold for medicinal/magic/pet purposes or used for snake charming. This unique epidemiological context has never been studied considering the interactions of humans, reptiles, and zoonotic pathogens. Thus, the aim of this study was to identify the parasites and pathogens present in blood and feces associated with handled reptiles in the markets of Marrakech to assess the risk of zoonotic transmission within the reptile-human interface. Privately owned reptiles (*n* = 118), coming from vendors or snake charmers, were examined and blood and feces sampled. DNA was extracted and molecular screening (cPCR, nPCR, qPCR, dqPCR) was performed aiming to identify potentially zoonotic pathogens (i.e., *Anaplasma*/*Ehrlichia* spp., *Rickettsia* spp., *Borrelia burgdorferi* sensu lato, *Coxiella burnetii*, *Babesia/Theileria* spp., *Cryptosporidium* spp., *Giardia* spp., *Leishmania* spp., Cestoda). Overall, 28.9% (34/118) of reptiles were positive for at least one pathogen. In blood, *Anaplasma* spp. were detected in four snakes, with two Montpellier snakes positive for *Anaplasma phagocytophilum*, while *Rickettsia* spp. were detected in one Mediterranean chameleon and four puff adders. *Leishmania tarentolae* was molecularly detected in a Mediterranean chameleon and a Montpellier snake. In feces, the *cox*1 gene generated a myriad of sequences for nematodes, cestodes, fungi and bacteria. Importantly, *Proteus vulgaris* was identified from a Mediterranean chameleon. *Cryptosporidium* spp. nPCR yielded a positive sample (i.e., *Cryptosporidium* sp. apodemus genotype I) from a Moroccan worm lizard, as well as for bacteria such as *Pseudomonas aeruginosa* in an Egyptian cobra, and *Morganella morganii* from a puff adder. Results from this study demonstrated the risk of zoonotic transmission of microorganisms and parasites present in blood and feces from reptiles that are brought to the souks in Marrakech, Morocco, to be sold for medicinal purposes or used for snake charming, being in direct and straight contact with humans.

## Author summary

The world-famous Marrakech souks (markets) are a unique and scarcely studied context where reptiles are in constant contact with humans and used for traditional medicine or for the ancient craft of snake charming. Hence, we aimed to identify the pathogens present in blood and feces of reptiles kept in the markets of Marrakech to assess the zoonotic risk. Animals from vendors or snake charmers were sampled. DNA was extracted and molecularly screened to identify potentially zoonotic pathogens. Overall, 28.9% of reptiles were positive for at least one pathogen. Importantly, we detected *Anaplasma* spp. from four snakes, *Rickettsia* spp. from Mediterranean chameleons and puff adders, and *Leishmania tarentolae* from snakes and chameleons. On the other hand, zoonotic bacteria such *Proteus vulgaris* were identified from a Mediterranean chameleon, *Pseudomonas aeruginosa* from Egyptian cobra, and *Morganella morganii* from puff adder. Thus, reptiles that are kept in the souks of Marrakech may play a role in the zoonotic transmission of pathogens and parasites, given the constant contact with humans.

## Introduction

Souk is an Arabic term which means “the market”, and in North Africa and the Middle East it is an important center, not only of commerce but of tradition, and culture of Arab-Islamic societies [1]. The origin of these important markets is believed to be strictly associated with the evolution and diffusion of the Islamic societies. Nonetheless, archeological documentation of ancient souks is scarce, yet records of souks date back from 3000 B.C. from Anatolian Persia [2]. In Morocco, the souks of Marrakech represent the largest and most famous markets, becoming in recent years not only the heart of Marrakech’s commerce, trade, and art, but also an important touristic hotspot [3]. Marrakech’s old city or *medina* concentrates the largest number of souks, which are near the enigmatic Jemaa-El-Fna square (also known as Jamâ-El-Fna or Djemaa-El-Fna square; literally: crossroads of the arts). This enigmatic square was founded in the 11^th^ century, and since then, it is a space of great complexity, involving Moroccan traditions, displayed through art, religion, music and gastronomy. Given its souks and its cultural heritage which has given the city the designation of World Heritage Site by UNESCO twice, Marrakech has become one of the most known tourist destinations in Morocco and North Africa [4]. Within Jemaa-El-Fna square, presence of monkeys and snakes is common [3]. Certainly, despite the charm and touristic attractiveness of the Souks, there is the risk of exploitation of wild animals [5,6]. Specifically, regarding reptiles’ presence in the souks of Marrakech, their use is associated to medicinal purposes or snake charming [6,7]. Indeed, the souks within the medina of Marrakech are full of live reptiles, mainly spur-thighed tortoises (*Testudo graeca*), Mediterranean chameleons (*Chamaeleo chamaeleon*), and occasionally Bell’s Dabb monitor lizards (*Uromastyx acanthinura*) and desert lizards (*Varanus griseus*), that are used with etnoherpetological purposes such as traditional medicine and magic [7]. Likewise, animal anatomical pieces are also commercialized with the same purposes being the desert lizard, Nile crocodile (*Crocodylus niloticus*), and the African rock python (*Python sebae*), both species being exotic to Morocco, as well as heads of the Egyptian cobra (*Naja haje*), present throughout the souks of Marrakech [6]. On the other hand, snake charming is still a prevalent craft in large cities of Morocco, being Marrakech the main center of this activity in the country, with descriptions of its existence since the late 1700s [8]. Snake charming is mainly practiced by a religious brotherhood called *Aissawa*, which claim to be immune to the snakes’ venom. Commonly used species for snake charming in the Jemaa-El-Fna square are the Egyptian cobra and the puff adder (*Bitis arietans*), followed by the horned viper (*Cerastes cerates*) and Moorish viper (*Daboia mauritanica*). Mildly to non-venomous species are also used such as the Montpellier snake (*Malpolon monspessulanus*), and to a lesser extent the horseshoe whip snake (*Hemorrhois hippocrepis*) [8]. Snakes used by charmers are generally captured (from April to October) and brought to the souks of Marrakech from the Atlantic belt of South-Western Morocco, where species of snakes (more than 27 species) thrive [8]. The overall welfare, husbandry and living conditions of animals within the souks are scarce. Animals are kept in crowded cages, with no water and generally surrounded by their own feces [5].

These unhygienic conditions, coupled with the constant handling and proximity to people, are driving factors for zoonotic pathogens’ transmission. Indeed, in this context, humans may be exposed to reptile-borne pathogens and reptile vector-borne diseases (RBVDs), given their proximity, and constant interaction [9,10]. Within the various transmission pathways, environmental contamination or oral-fecal transmitted pathogens have greater possibilities to infect people *via* reptile handling. Besides *Salmonella*, many other bacteria and parasites can be transmitted [9,11]. However, there are no studies assessing the prevalence of these pathogens associated with the human-reptile interface. Moreover, other zoonotic pathogens associated to reptiles (e.g., *Anaplasma*, *Borrelia*, *Rickettsia*) are transmitted by vectors (i.e., mosquitoes, ticks, and sand flies) [10]. Previous studies in Moroccan herpetofauna have addressed *Salmonella* [12], and ticks [13] from tortoises, as well as herpetophilic sand flies’ species and their potential role in the transmission of leishmaniases [14]. In addition, ecological studies have been performed on the prevalence and distribution of hemoparasites, such as *Hepatozoon* spp. associated to Moroccan reptiles [15,16], or on the prevalence of parasitic fauna of endemic species of reptiles [17]. Conversely, there are no studies addressing the human-reptile-parasite interface in the souks of Marrakech context, or neither on the zoonotic pathogens associated to highly handled animals such as snakes used by charmers.

Thus, the aim of this study was to identify the microorganisms and parasites present in blood and feces from reptiles (i.e., lizards, tortoises, and snakes) kept in the Jemaa-El-Fna square to assess the prevalence of pathogens of zoonotic concern.

## Methods

### Ethics statement

The study was conducted in accordance with all applicable international, national, and/or institutional guidelines for the care and use of animals. Protocols of reptile sampling were authorized by the *Office National de Sècurité Sanitaire des Produits Alimentaires* from the Kingdom of Morocco (Approval number 23355ONSSA/DIL/DPIV/2022).

### Animal examination and sampling

**Fig 1:**
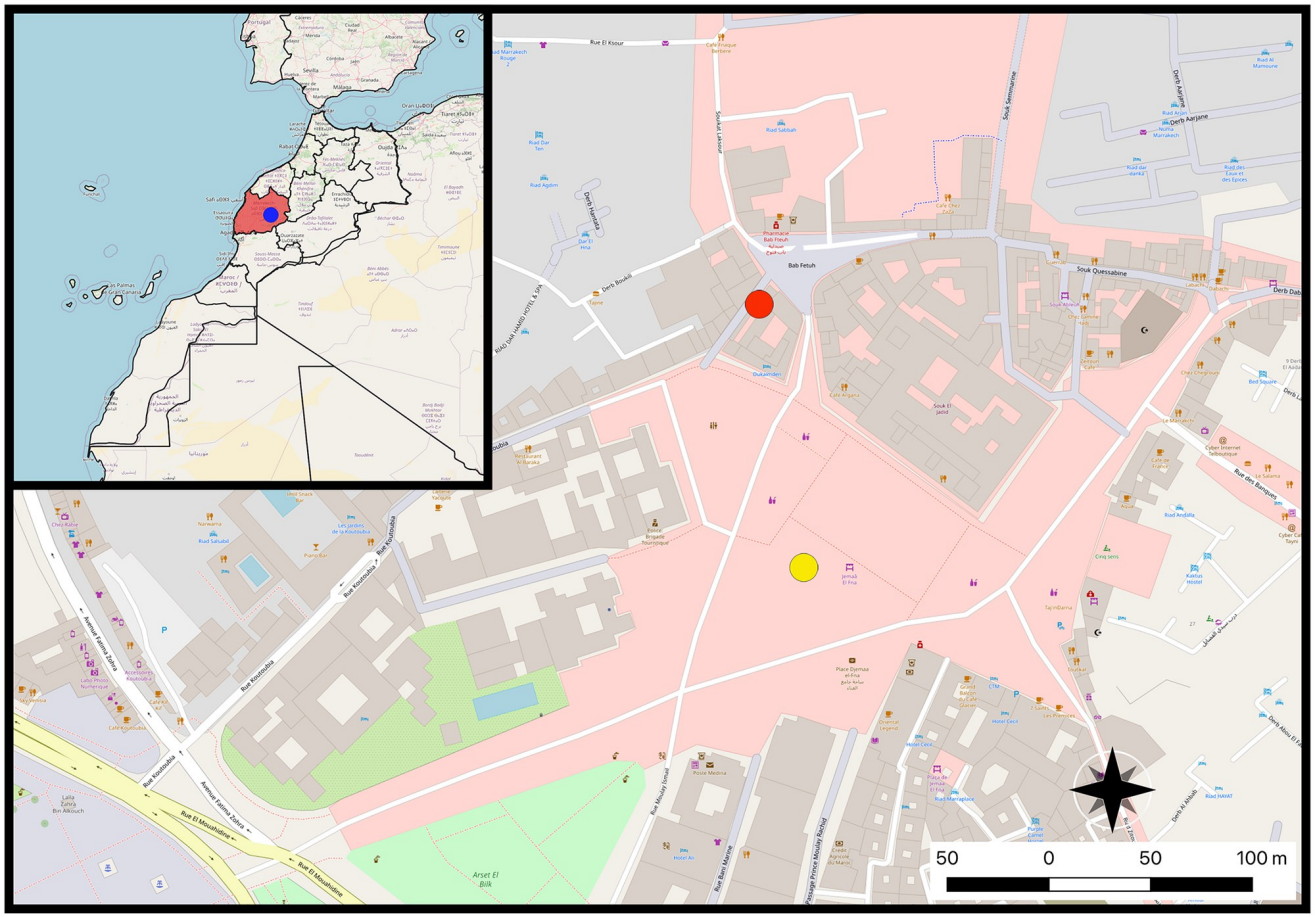
Map of the Jemaa-El-Fna square, Marrakech, Morocco. Blue circle represents Marrakech municipality; Yellow circle represents site where snakes used by charmers are displayed, and site where reptiles are sold by vendors is represented by a red circle. Map prepared using QGIS software—Buenos Aires version (link of the XYZ tile: https://tile.openstreetmap.org).

**Fig 2:**
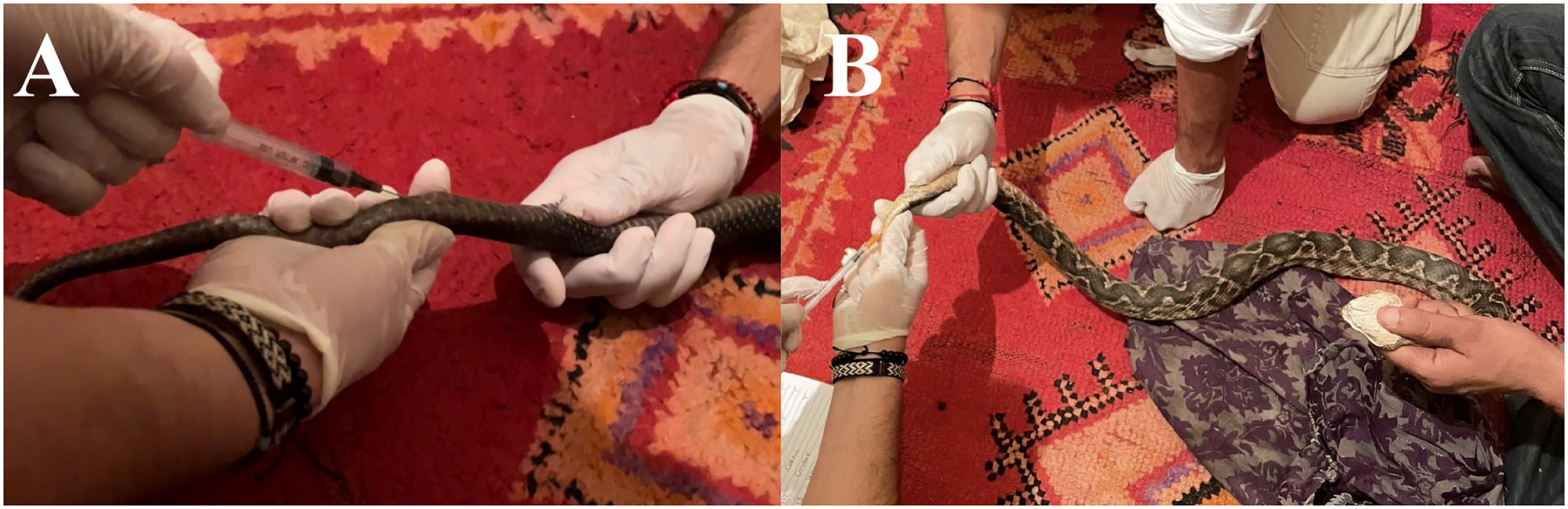
Blood draw from the ventral coccygeal vein of (a) Egyptian cobra (*Naja haje*) and (b) puff adder (*Bitis arietans*).

In October 2022, reptiles kept within the proximities of Jeema-El-Fna square of Marrakech (Fig 1) were examined, morphologically identified to species level using reference keys or checklists [18–20], and sampled after authorization of their owners (i.e., vendors or snake charmers; Fig 1). A blood sample (Fig 2) was obtained from each animal. Blood (~100 μl to 1ml) was drawn from snakes and lizards using the ventral coccygeal vein, whereas tortoises’ blood was drawn from the subcarapacial sinus. Blood was divided in Whatman FTA Cards and 1.5 ml Eppendorf tubes which were later stored at -20 °C. Cloacal swabs were performed from all animals and stored at -20 °C. Blood smears were performed from all animals and then assessed for the presence of hemoparasites [21] using Diff-Quik stain [22]. Smears were rinsed in tap water to remove excess stain and later evaluated using an optical microscope (LEICA DM LB2, Germany).

### Molecular screening of pathogens

DNA was extracted from individual blood samples (*n* = 112) and cloacal swabs (*n* = 102) using a commercial kit (QIAamp DNA Mini Kit, Qiagen, Hilden, Germany), according to the manufacturer’s instructions and analyzed for the detection of different microrganisms and parasites (see below). Details regarding cPCR and qPCR protocols are reported in Table 1. All cPCR products were examined on 2% agarose gel stained with GelRed (VWR International PBI, Milan, Italy) and visualized on a GelLogic 100 gel documentation system (Kodak, New York, USA). Amplicons were then purified and sequenced in both directions using the same primers as for PCRs, by the Big Dye Terminator version 3.1 chemistry in a 3130 Genetic Analyzer (Applied Bio-systems, Foster City, CA, USA). Sequences were edited and analyzed using Geneious software version 9.0 (Biomatters Ltd., Auckland, New Zealand) [23] and compared with those available in the GenBank database by the Basic Local Alignment Search Tool (BLAST; http://blast.ncbi.nlm.nih.gov/Blast.cgi) for species identification. Nested PCRs were performed for *Giardia* spp. and *Cryptosporidium* spp. as follows. For *Giardia*, a nested PCR amplifying partial triosephosphate isomerase (*tpi*) gene (532 bp) was used, to also detect all known assemblages [24,25]. Another nPCR was used to detect *Cryptosporidium* spp., targeting a fragment of the 18S rRNA gene [26].

**Table 1:** Pathogens screened in this study by conventional (c) and quantitative (q) PCR, with target genes, primers, probes nucleotide sequences and fragment length.

	Pathogens	Target gene	Primers	Sequence (5′−3′)	Fragment length (bp)	References
**cPCR**	*Anaplasma*/*Ehrlichia* spp.	16S rRNA	*EHR-16SD*	GGTACCYACAGAAGAAGTCC	345	[27]
			*EHR-16SR*	TAGCACTCATCGTTTACAGC		
	*Borrelia burgdorferi* sensu lato	Flagellin	*FLA1*	AGAGCAACTTACAGACGAAATTAAT	482	[28]
			*FLA2*	CAAGTCTATTTTGGAAAGCACCTAA		
	*Rickettsia* spp.	*glt*A	*CS-78F*	GCAAGTATCGGTGAGGATGTAAT	401	[29]
			*CS-323R*	GCTTCCTTAAAATTCAATAAATCAGGAT		
	Spotted Fever Group Rickettsiae	*omp*A	*Rr190*.*70F*	ATGGCGAATATTTCTCCAAAA	632	[30]
			*Rr190*.*701R*	GTTCCGTTAATGGCAGCATCT		
	*Babesia/Theileria* spp.	18S rRNA	RLB-F	GAGGTAGTGACAAGAAATAACAATA	460–520	[31]
			RLB-R	TCTTCGATCCCCTAACTTTC		
	*Coxiella burnetii*	IS1111a	Trans-1	TATGTATCCACCGTAGCCAGT	687	[32]
			Trans-	CCCAACAACACCTCCTTATTC		
	Cestodes/Nematodes	*cox*1	JB3	TTTTTTGGGCATCCTGAGGTTTAT	400	[33]
			JB4.5	TAAAGAAAGAACATAATGAAAATG		
**qPCR**	*Leishmania* spp.	kinetoplast	LEISH-1	AACTTTTCTGGTCCTCCGGGTAG	120	[34]
			LEISH-2	ACCCCCAGTTTCCCGCC		
			Probe	6-FAM-AAAAATGGGTGCAGAAAT-MGB		
	Duplex *Leishmania*	ITS1	L.i.t. -ITS1-F	GCAGTAAAAAAAAGGCCG	150	[35]
			L.i.t. -ITS1-R	CGGCTCACATAACGTGTCGCG		
			Probe L.t.	6-FAM-CACGCCGCGTATACAAAAACAC-MGB		
			Probe L.i.	VIC-TAACGCACCGCCTATACAAAAGCA-MGB		
	*Giardia duodenalis*	SSU	Giardia-80F	GACGGCTCAGGACAACGGTT	62	[36]
			Giardia-127R	TTGCCAGCGGTGTCCG		
			Giardia-105	Fam-5′- CCCGCGGCGGTCCCTGCTAG-3′-Tamra		

### Phylogenetic analyses

Rickettsial *gltA* as well as 16S rRNA sequences from *Anaplasma* spp. were separately aligned against those closely related species available from GenBank database using the ClustalW application within MEGA7 software [37]. The Akaike Information Criterion (AIC) option in MEGA7 was used to establish the best nucleotide substitution model adapted to each sequence alignment. Tamura-Nei model with a invariant sites (I) [38] was used to generate the *gltA* and the 16S rRNA trees. A maximum likelihood (ML) phylogenetic inference was used with 2000 bootstrap replicates to generate the phylogenetic tree in MEGA7. Homologous sequences of *Rickettsia* were used as outgroup to root the trees, including the *glt*A sequences from *Rickettsia belli* and *Rickettsia canadensis* (AB297809), and the 16S sequence of *Rickettsia parkeri* (NR118776).

## Results

### Reptile examination and sampling

**Fig 3:**
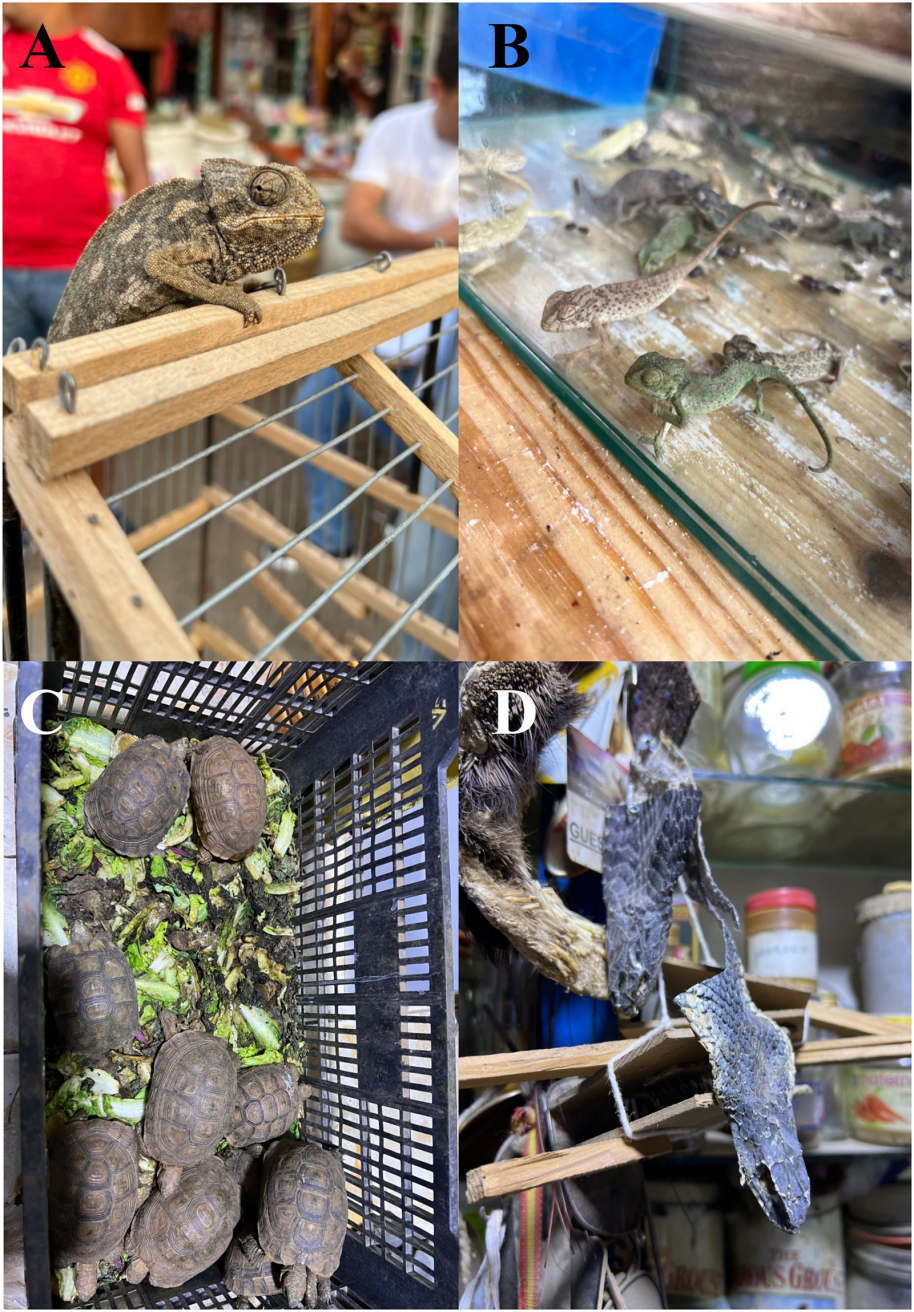
Reptiles commercialized in the souks of Marrakech, a) adult Mediterranean chameleon (*Chamaeleo chamaeleo*), b) newborn Mediterranean chameleon, c) spur-thighed tortoises (*Testudo graeca*), d) Egyptian cobra (*Naja haje*) dried heads.

**Fig 4:**
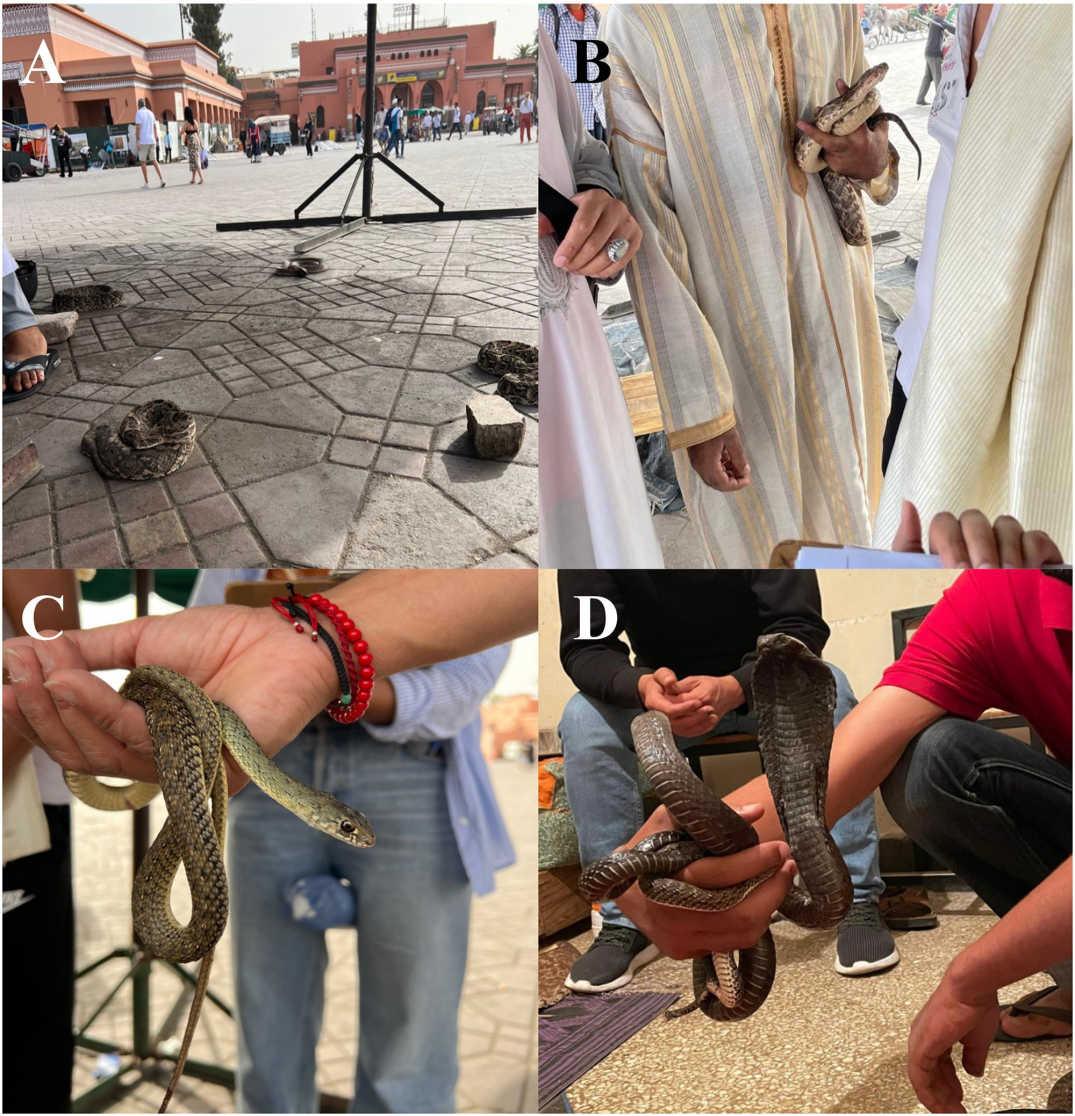
Snakes used by charmers in the Jemaa-El-Fna square. a) puff adders, b) charmer handling a puff adder, c) charmer with a Montpellier snake (*Malpolon monspessulanus*), d) charmer with an Egyptian cobra.

Overall, 118 reptile specimens were examined and screened represented by two orders [Squamata (Amphisbaenia with one species, Sauria with two species in two families, Ophidia with five species in three families), and Testudines (one species); Table 2. Species of reptiles commercialized in the markets were from Amphisbaenia, Sauria and Testudines (Fig 3). In addition, 68 snake specimens used by charmers were screened represented mainly by Montpellier snakes, followed by puff adders, Egyptian cobras, eastern Montpellier snake, and one individual of horseshoe whip snake (Table 2). Two of the before mentioned species are considered highly venomous (i.e., puff adder, Egyptian cobra), whereas both species of *Malpolon* are considered mildly venomous rear-fanged (i.e., opisthoglyphous) snakes. Instead, the horseshoe whip snake is a non-venomous aglyphous snake (Fig 4).

**Table 2:** Species of reptiles (scientific and common names) sampled along with numbers and type of owners.

Owned by	Order (Suborder)	Family	Species (n)	Common name
Vendors n = 50	Squamata (Sauria)	Chamaeleonidae	*Chamaeleo chamaeleon* (30)	Mediterranean chameleon
		Scincidae	*Eumeces algeriensis* (1)	Algerian skink
	Squamata (Amphisbaenia)	Blanidae	*Blanus mettetali* (5)	Moroccan worm lizard
	Testudines (Cryptodira)	Testudinidae	*Testudo graeca* (14)	Spur-thighed tortoises
Charmers n = 68	Squamata (Serpentes)	Viperidae	*Bitis arietans* (20) ***	Puff adder
		Colubridae	*Hemorrhois hippocrepis* (1) *	Horseshoe whip snake
		Psammophiidae	*Malpolon insignitus* (9) **	Eastern Montpellier snake
			*Malpolon monspessulanus* (28) **	Montpellier snake
		Elapidae	*Naja haje* (10) ***	Egyptian cobra
**Total**			118	

* Non-venomous

** Mildly venomous

*** Highly venomous

### Pathogen detection

**Fig 5:**
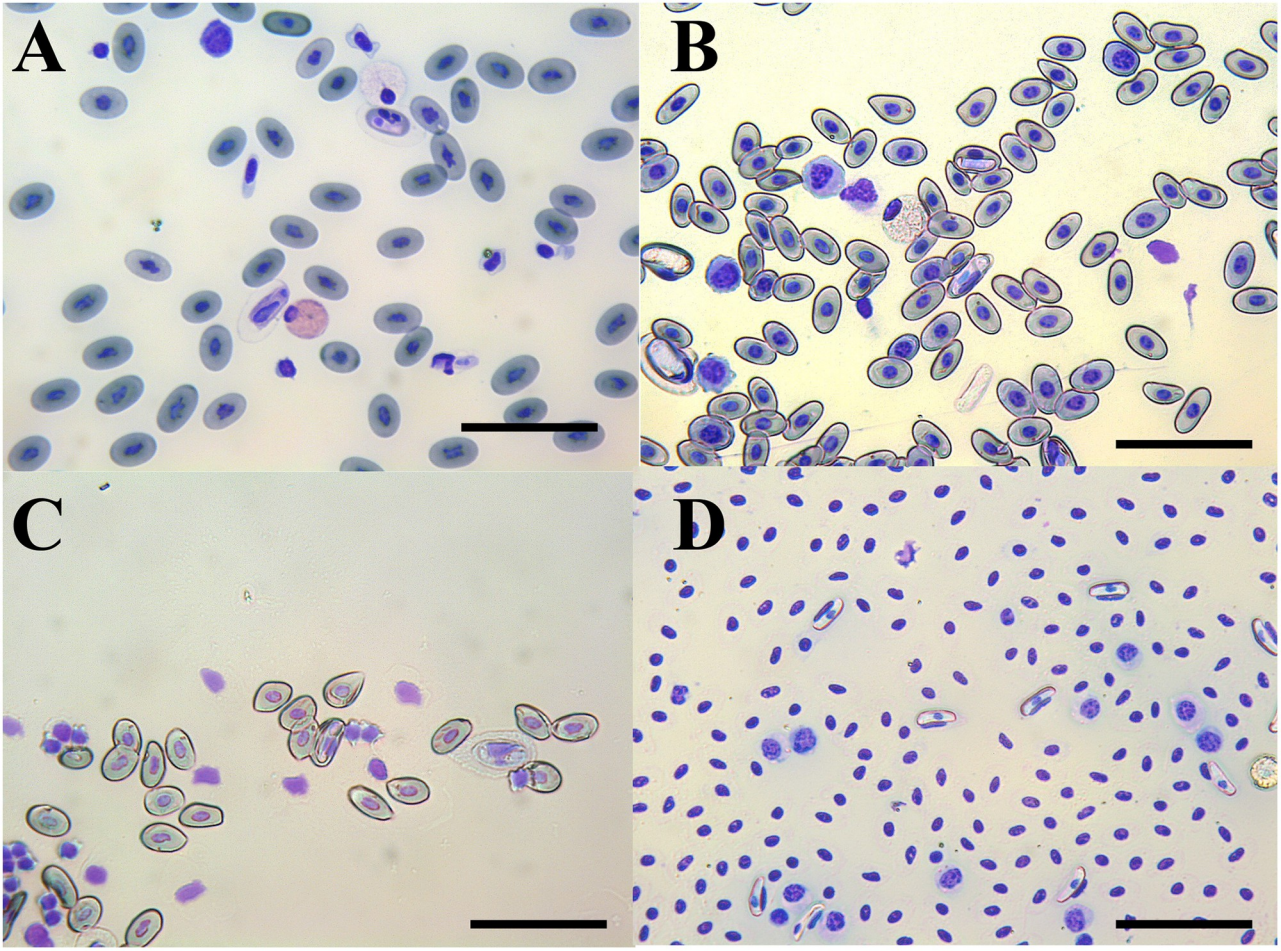
Gametocytes of hemogregarines in erythrocytes of snakes. a) gamonts in erythrocytes of puff adder, b) gamonts in erythrocytes of horseshoe whip snake (*Hemorrhois hippocrepis*), c) gamonts in erythrocytes of Montpellier snake, d) gamonts in erythrocytes of Egyptian cobra. Scale bars 50μm.

**Fig 6:**
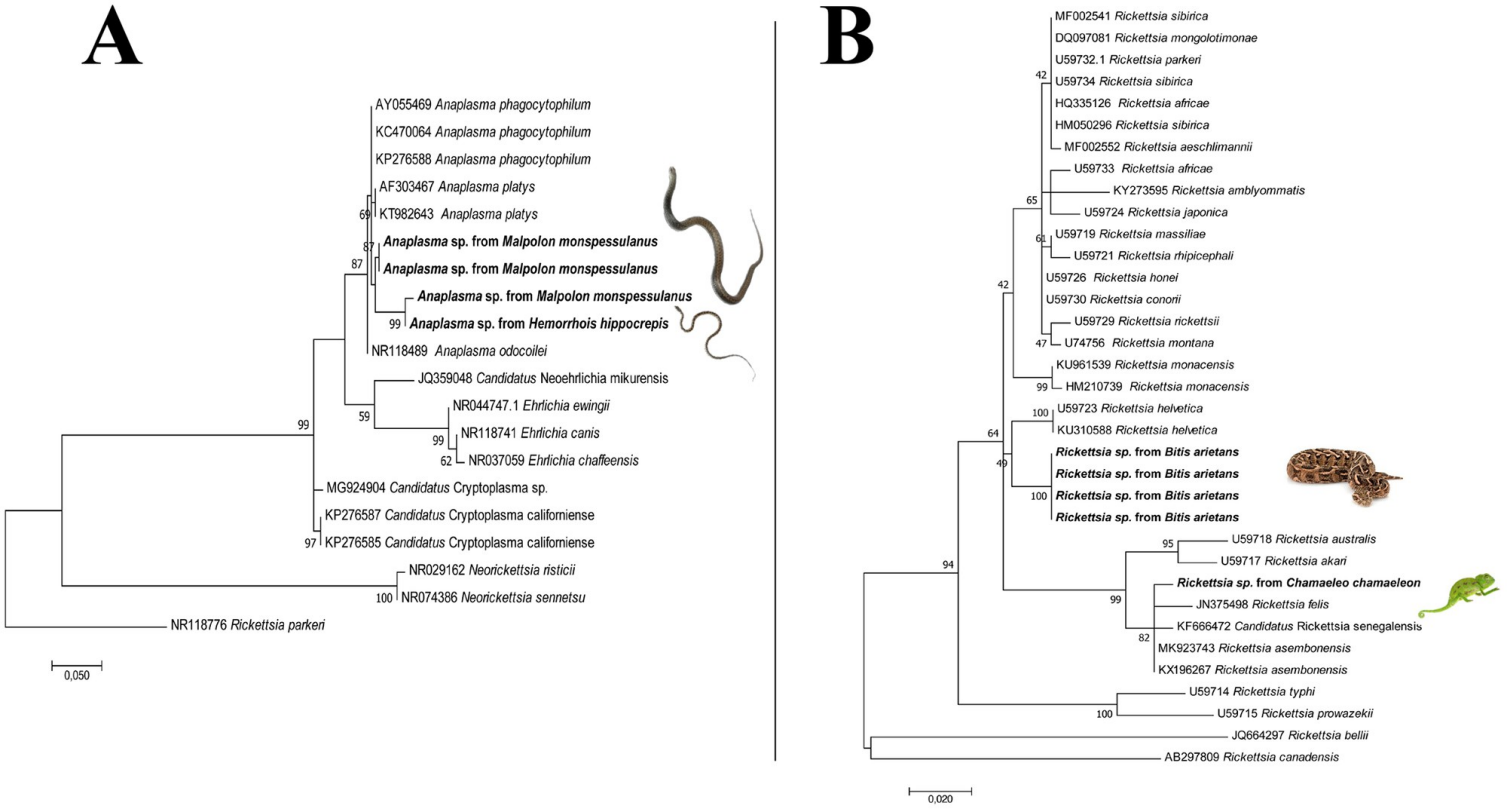
Maximum-likelihood phylogenetic trees of (a) 16S rRNA sequences of Anaplasmataceae and of *gltA* (b) genes of *Rickettsia* spp. Bootstrap values (>40%) are shown near the nodes. *Rickettsia parkeri* (a) *Rickettsia belli*, *Rickettsia canadensis* (b) were used as outgroups. Scale bar indicates nucleotide substitution per site. Sequences of this study are in bold.

Positive blood smear observation yielded the presence of hemogregarines’ gametocytes in 22% of examined individuals (26/118; Fig 5). Gamonts were only observed in the five species of snakes (Table 3). Overall, 28.9% (34/118) of examined reptiles were positive to at least one molecularly identified pathogen, being 9.3% (11/118) animals owned by vendors and 19.5% (23/118) snakes used by charmers. Molecular screening of pathogens in blood rendered positive results for *Anaplasma*/*Ehrlichia* spp., *Rickettsia* spp. (*glt*A), *Babesia/Theileria* spp., and *Leishmania* spp. (ITS) (Table 3), whereas all samples were negative for *Borrelia burgdorferi* sensu lato, Spotted Fever Group Rickettsiae or *Coxiella burnetii*. Specifically, four snakes (3.4%) were positive for *Anaplasma* spp., with two Montpellier snakes positive to *Anaplasma phagocytophilum* (i.e., 99.7% nucleotide identity with MT126499) of horses from Turkey. All generated sequences of *Anaplasma* sp. clustered within the species of *Anaplasma* (*A*. *phagocytophilum*, *Anaplasma platys*, *Anaplasma odocoilei*) with high bootstraps values (i.e., 87%) (Fig 6A). On the other hand, 16S rRNA target gene also amplified in a puff adder for the endosymbiont *Candidatus* Midichloria mitochondrii (100.00% nucleotide identity with EU780455 of *Cimex lectularius*). In addition, 4.2% of reptiles (5/118; four puff adders and one Mediterranean chameleon) were positive for *Rickettsia* spp. The four puff adders were positive to *Rickettsia asiatica* (97.7% homology with AP019563), and the Mediterranean chameleon to *Candidatus* Rickettsia asembonensis (100% nucleotide identity with MK923743 of *Ctenocephalides canis*). Phylogenetic inference clustered four sequences of *Rickettsia* sp. from puff adders with *Rickettsia helvetica*, whereas *Rickettsia* sp. sequence from a Mediterranean chameleon clustered with *R*. *asembonensis*, *Rickettsia felis* and *Candidatus* Rickettsia senegalensis (Fig 6B). Furthermore, despite the high prevalence in blood smears, only three sequences were obtained for *Hepatozoon* spp. in a Mediterranean chameleon, a Montpellier snake, and a puff adder (99.9% nucleotide identity with KC696565), previously detected in *Psammophis schokari* snake form North Africa (Table 3). Additionally, the dqPCR detected two animals (1.7%) positive to *Leishmania tarentolae* i.e., a Mediterranean chameleon (*ct* 35.31) and a Montpellier snake (*ct* 34.56).

**Table 3:** Vector-Borne pathogens and oral-fecal pathogens detected in reptiles by blood smear or molecular identification.

Species of reptile (infected/total)	Vector-Borne pathogen					Oral-fecal pathogen	
	Gametocytes present in blood smears	cPCR (16S rRNA)	cPCR (18S rRNA)	cPCR (*gltA*)	dqPCR (ITS1)	cPCR (*cox*1)	nPCR (18S rRNA)
*Chamaeleo chamaeleon* (4/30)			(1) KC696565 *Hepatozoon* sp. (99.8%)	(1) MK923743 *Rickettsia asembonensis* (100%)	(1) *Leishmania tarentolae* (*ct* 35.31)	(1) CP054157 *Proteus vulgaris* (92%)	
*Eumeces algeriensis* (0/1)							
*Blanus mettetali* (1/5)							(1) MH912997 *Cryptosporidium* sp. apodemus genotype I (92%)
*Testudo graeca* (6/14)						(1) HQ317434 *Enterobius vermicularis* (89%) (1) MH427272 *Syphacia obvelata* (86%) (2) KJ939328 *Trypanoxyuris atelis* (83%) (2) KU285486 *Trypanoxyuris multilabiatus* (83%)	
*Bitis arietans* (18/20)	(10/20)	(1) EU780455 *Candidatus* Midichloria mitochondrii (100%)	(1) KC696565 *Hepatozoon* sp. (99.8%)	(4) AP019563 *Rickettsia asiatica* (97.7%)		(1) KY498478 *Neurospora crassa* (91.1%)	(1) CP032295 *Morganella morganii* (98.5%)
*Hemorrhois hippocrepis* (1/1)	(1/1)	(1) MK041546 *Anaplasma* sp. 98.79%					
*Malpolon insignitus* (3/9)	(1/9)					(1) KR780799 *Penetrocephalus ganapattii* (83%) (1) KY498478 *Neurospora crassa* (92%)	
*Malpolon monspessulanus* (23/28)	(13/28)	(1) MW790941 *Anaplasma* sp. (99.69%) (2) MT126499 *Anaplasma phagocytophilum* (99.7%)	(1) KC696565 *Hepatozoon* sp. (99.8%)		(1) *Leishmania tarentolae* (*ct* 34.56)	(1) MH463505 *Mesocestoides* sp. (83%) (1) MH427272 *Syphacia obvelata* (84%) (2) KJ939328 *Trypanoxyuris atelis* (83%)	(1) CP064828 *Morganella morganii* (98.5%)
*Naja haje* (3/10)	(1/10)						(1) MK394156 *Diutina catenulate* (99.8%) (1) CP050335; *Pseudomonas aeruginosa* (99.5%)
**Total** (59/118)	(26/118)	(5/118)	(3/118)	(5/118)	(2/118)	(14/118)	(5/118)

Conversely, molecular screening of pathogens in feces revealed positive results for a great variety of agents. Particularly, a myriad of sequences were obtained with the *cox*1 gene, from nematodes and cestodes, to fungi and bacteria. Nematodes were detected in eight (6.7%) spur-thighed tortoises and two (1.7%) Montpellier snakes. Sequences generated were similar (83 to 89%) to *Enterobius* (HQ317434), *Syphacia* (MH427272) and *Trypanoxyuris* (KJ939328) genera. Moreover, cestodes sequences were retrieved from two snakes (eastern Montpellier snake and Montpellier snake) represented by *Mesocestoides* (MH463505) and *Penetrocephalus* (KR780799) genera (83% of nucleotide identity). Conversely, *Neurospora crassa* fungi (KY498478; 92% of nucleotide identity) was detected in two snakes (puff adder and eastern Montpellier snake) and *Proteus vulgaris* bacteria (CP054157; 92% nucleotide identity) in Mediterranean chameleon.

Lastly, *Cryptosporidium* spp. 18S rRNA gene nPCR revealed positive samples for *Cryptosporidium* sp. apodemus genotype I (MH912997; 92% of nucleotide identity) in one Moroccan worm lizard. Also, the nPCR yielded positive results for bacteria such as *Pseudomonas aeruginosa* (CP050335; 99.5% of nucleotide identity) in one Egyptian cobra, and *Morganella morganii* (CP032295; CP064828; 98.5% of nucleotide identity) in one puff adder and one Montpellier snake. Additionally, fungi (*Diutina catenulate*—MK394156; 99.8% of nucleotide identity) were detected in an Egyptian cobra. Representative sequences herein generated were deposited in GenBank (accession numbers OQ633057 to OQ633061 for *gltA*; OQ630499 to OQ630503 for 16S rRNA; OQ632771 to OQ632773 for 18S rRNA; OQ672452, OQ695490 and OQ695491 for *cox*1 and 18S rRNA).

## Discussion

A wide diversity of microorganisms and parasites were detected from reptiles that are brought to the souks of Jemaa-El-Fna square in Marrakech. Importantly, while some of the identified parasites are specific of reptiles and are non-pathogenic (i.e., *Hepatozoon*), many of the bacteria, fungi and parasites detected have a zoonotic potential. Data demonstrated the presence of vector-borne (e.g., *Anaplasma*, *Rickettsia*, *Leishmania*), as well as orally transmitted zoonotic pathogens (i.e., *Cryptosporidium*, *Pseudomonas*, *Morganella* and *Diutina*) associated to reptiles thus representing a risk of infection to vendors and charmers of the souks of Marrakech.

Overall, the 118 reptile specimens examined fairly represent the typical Moroccan herpetofauna associated to the practices of traditional medicine, magic, and snake charming [5]. As observed in previous studies, Mediterranean chameleons and spur-thighed tortoises are heavily commercialized in the markets, being captured in large numbers from the wild [39,40], whereas Moroccan worm lizards are rarely seen in this context [6]. Indeed, previous studies showed that one of the major threats for wild populations of chameleons and tortoises is the non-commercial collection, which seems a common activity in Morocco [40]. In this sense, many day-buyers of the souks purchase chameleons and specially tortoises (i.e., 55% of the Moroccan population has tortoises in their households), to keep as pets [40,41]. Similarly, species of snakes used by charmers were also represented by the common and more attractive species, particularly Egyptian cobras, which are considered the most profitable species given its unique display of warning behavior [7,8]. Moreover, puff adders and species of *Malpolon* are also widely used, being the Montpellier snake (although mildly venomous) handed to tourists for taking pictures [7]. Considering vendors and snake charmers, the latter represent an understudied work category at risk given the possibility of snakebites, which represents a neglected tropical disease that affects more than 2.7 million people annually, killing up to 138,000 people, as well as leaving more than 400,000 people with disabilities [42]. In Morocco, snakebites are an important public health issue, mainly in the central regions. However, studies show that up to 86% of the snakebites were reported from males in rural areas caused by vipers or colubrid snakes [43–45]. Despite the constant contact with venomous animals, snakebite envenomation in charmers seems to be underreported, most likely given the possibility of charmers losing their livelihood if they report snakebites [8,46]. Moreover, this study represents the first screening for microorganisms and parasites in reptiles that are in constant contact with humans, on many occasions under unsanitary conditions, favoring transmission of zoonotic pathogens [5]. Indeed, molecular screening allowed to identify not only pathogens that can be transmitted by environmental/oral contamination [9], but also those transmitted by vectors [10], and fungi and bacteria that may also be of zoonotic concern, highlighting the importance of performing molecular screening of wild animals in anthropized environments.

Cytological and molecular identification of hemogregarines, most likely *Hepatozoon*, is in accordance with former studies in the same geographical area of the Mediterranean Basin [47]. Indeed, *Hepatozoon* infection in North African snakes is common, with results of this study showing higher infection rates (22%) compared to global prevalence of 8% previously reported [47]. However, even if *Hepatozoon* spp. are considered non-pathogenic, with no zoonotic relevance, further ecological studies are encouraged to better understand the circulation of species of hemogregarines and their transmission routes in reptiles. Conversely, molecular detection of *Anaplasma*, chiefly *A*. *phagocytophilum*, underlines the potential role reptiles, in particular snakes, may have as amplifying reservoirs of the causative agent of granulocytic anaplasmosis [10]. Indeed, in other ecological scenarios (i.e., Northern California, US), snakes were found PCR positive to human-derived *A*. *phagocytophilum* [48]. In Morocco, *A*. *phagocytophilum* is prevalent in canine, small ruminant, and human populations mainly in the northern regions [49–51], though molecular positivity of *Anaplasma* in Moroccan colubrid snakes is an unprecedented finding. The fact that these animals were captured from the wild, coupled by the finding of *Candidatus* Midichloria mitochondrii endosymbiont in a puff adder indicates the exposure to ticks that may vector *Anaplasma*. In fact, *Candidatus* Midichloria mitochondrii may be used in vertebrate hosts to assess the exposure to ticks [52]. Furthermore, this is the first molecular identification of *Candidatus* Midichloria mitochondrii in reptiles. In addition, evidence of tick exposure of wild reptiles is demonstrated by the molecular detection of *Rickettsia* spp., as previously detected in the Mediterranean basin [53], as well in the Neotropics [54], Africa and Asia [55]. Molecular identification of *Rickettsia* spp. in blood of vertebrate hosts is an uncommon finding, yet the prevalence of *Rickettsia* spp. herein reported was higher (4.1%) than in previous studies (i.e., 3.1%), in the northern Mediterranean basin [53]. Thus, further studies are needed to assess the prevalence of pathogenic *Rickettsia* spp. in ticks associated with reptiles, considering that in Morocco four pathogenic *Rickettsia* were detected from ticks from domestic animals and the environment [43]. Also, considering that *Rickettsia monacensis* was detected in *Ixodes ricinus* from Morocco [56] as well as in Italy from the same species of ticks associated to lizards [53], tick surveys of wild captured reptiles are advocated to assess the risk of vector-borne transmission of pathogenic rickettsiales.

The vector-borne protozoan *L*. *tarentolae* was herein identified for the first time in the Mediterranean chameleon and in the Montpellier snake, further suggesting the wide distribution of this reptile-associated *Leishmania* sp. throughout the Mediterranean Basin. Indeed, previous studies identified *L*. *tarentolae* from lizards and geckos [57], and data herein presented suggest that snakes may also be competent hosts. Furthermore, the vector of this *Leishmania* species, *Sergentomyia minuta*, has a widespread geographical distribution in Morocco, mainly in the northern and central regions where visceral and cutaneous leishmaniases are endemic [14]. In addition, evidence of anthropophilic feeding behavior of *S*. *minuta* [58], as well as the mammal (humans and canines) exposure to *L*. *tarentolae* [59,60], coupled with the sympatric occurrence of different species of *Leishmania* in Morocco (*L*. *infantum*, *L*. *major*, *L*. *tarentolae*, *L*. *tropica*) [61], further complicates the epidemiological picture of cutaneous and visceral leishmaniasis in the country. Hence, epidemiological surveys assessing the prevalence of *Leishmania* in sand flies, mammalian and reptilian hosts are necessary to elucidate the real status and interaction of *Leishmania* spp. and the possible infections, in previously considered non-permissive hosts and vectors.

On the other hand, molecular screening of fecal swabs performed herein enabled to detect not only targeted parasites (i.e., cestodes, nematodes, protozoa), but also allowed to identify pathogenic bacteria and fungi. Nonetheless, coprological surveys of reptiles are further advocated for a complete morpho-molecular identification of parasites. Indeed, nematode sequences herein generated had low nucleotide identity, which only allowed to identify these parasites to family level (i.e., Oxyuridae) most of them non-pathogenic specific of reptiles, except for *Enterobius* spp. Importantly, although the sequence of *Enterobius vermicularis* from a spur-thighed tortoise generated herein had a 92% nucleotide identity with human-derived *E*. *vermicularis* from Greece, previous studies identified this human oxyiurid species in the same tortoise species from Algeria [62]. Hence, tortoises, in the souk context, could act as potential source of oxyurid infections for humans, which therefore should be monitored [63]. Moreover, apart from reptile-specific to low-pathogenic cestodes and innocuous fungi, *P*. *vulgaris* was identified from a Mediterranean chameleon in the souk. As this species is used for medicinal/magic purposes, or as pets, the presence of potentially pathogenic foodborne *P*. *vulgaris* highlights the risk of zoonotic infection [64,65]. The identification of Gram-negative bacteria such as *P*. *vulgaris* in reptiles is a common finding in the cloaca, as reptiles are healthy carriers and spreaders of this potentially zoonotic pathogen [65].

Finally, the *Cryptosporidium* spp. 18S rRNA sequence analysis revealed not only a species of *Cryptosporidium* from a Moroccan worm lizards kept on a small market stand, but also pathogenic bacteria and fungi. Zoonotic *Cryptosporidium* species (i.e., *C*. *muris*, *C*. *parvum*, *C*. *tyzzeri*) can be found in snakes that have ingested infected rodents [66]. Ophidians are not infected but they might excrete the ingested oocysts, contaminating the environment. Conversely, the detection of a rodent genotype of *Cryptosporidium* in an insectivorous reptile such as the Moroccan worm lizard, could indicate true infection or natural exposure in the wild or in the market setting [67]. Another option, would be contamination *via* insect ingestion, as suggested in pet leopard geckos infected with *C*. *parvum*, which has been verified to be mechanically transmitted by flies carrying infectious oocysts [67,68]. However, pathogenicity of *Cryptosporidium* sp. apodemus genotype I is still unknown [69]. Bacteria and fungi identified with the *Cryptosporidium* nPCR were all potentially zoonotic, thus posing a particular risk for snake charmers and tourists. For instance, aerobic and facultative species of bacteria such as *P*. *aeruginosa* are potentially pathogenic to snakes as well as to humans [70]. Likewise, *Morganella morganii* is commonly present in snakes cloacal and oral cavity [71]. Thus, snakebites from venomous or non-venomous snakes not only represents a potential life-threatening risk *per se*, but it also a pathway for secondary bacterial infections. On the other hand, pathogenic yeasts as *D*. *catenulate* from a mildly venomous Montpellier snake, is a potential risk to snake charmers and tourists handling this species of snake. This species of fungi, formerly known as *Candida catenulata*, has been associated to superficial and invasive infections in both humans and animals [72].

## Conclusions

Data herein presented highlighted the potential risk of zoonotic infection with parasites, bacteria and fungi associated to reptiles kept, handled, and used in the souks of the Jemaa-El-Fna square in Marrakech, Morocco. Indeed, reptiles sold for medicinal purposes or used for snake charming, thus in direct and constant contact with humans, harbor zoonotic and pathogenic agents such as those belonging to the genera *Anaplasma*, *Rickettsia*, *Cryptosporidium*, *Pseudomonas*, *Morganella* and *Diutina* that may be transmitted through vectors, orally, or even snakebites. Thus, snake charmers that handle these snakes are at risk of contamination. Hence, studies under the One-Health approach are advocated to better understand the prevalence, occurrence, and epidemiological cycle of potentially shared pathogens in this unique context, that is the fascinating souk of Marrakech.
